# The playing position significantly influences return to sports and recurrences after an arthroscopic Bankart repair in competitive rugby players

**DOI:** 10.1177/1758573221993089

**Published:** 2021-02-17

**Authors:** Ignacio Pasqualini, Luciano Andrés Rossi, Franco Luis De Cicco, Ignacio Tanoira, Ignacio Alonso Hidalgo, Santiago Bongiovanni, Diego Hernán Giunta, Maximiliano Ranalletta

**Affiliations:** 37533 Hospital Italiano de Buenos Aires, Buenos Aires, Argentina

**Keywords:** Arthroscopic Bankart repair, shoulder instability, rugby

## Abstract

**Background:**

The purpose of our study was to investigate the influence of the different rugby playing positions on return to sports, functional outcomes, and recurrences after an arthroscopic Bankart repair.

**Methods:**

A total of 88 rugby players were treated for anterior shoulder instability in our institution between 2010 and 2018. Functional outcomes, return to sports, recurrences, complications, and revisions rates were evaluated according to the playing position.

**Results:**

Overall, 73.8% of the patients returned to rugby and 60% returned at the same level as before the injury. The tight forwards and outside backs experienced a significant decrease in their competitive level after surgery, and showed the lowest functional outcomes. The tight forwards and outside backs showed a statistically significant increase in recurrence and revision rates, and an OR for recurrence of 12.8 and 9.6, respectively.

**Discussion:**

The playing position significantly influenced return to sports and recurrences after an arthroscopic Bankart repair in competitive rugby players. Specifically, the tight forwards and outside backs have returned to a lower level than they had before surgery, showed the lowest functional outcomes, and a significant increase in recurrences and revisions rates than the other groups.

## Introduction

Rugby is a popular collision sport in which shoulder injury incidence is much more prevalent than in other sports.^1^ Of all injuries, shoulder injuries have been documented to account for 9% to 11% among rugby players.^1^ Although some studies in the literature have shown that acromioclavicular dislocation is the most frequent injury, anterior shoulder dislocation is considered the most severe injury because it leaves rugby players the longest time out of competition and it has the highest recurrence rate.^1–3^

In the current literature, several studies have shown the effectiveness of the arthroscopic Bankart repair (ABR) regarding sports outcomes.^4,5^ However, these results could be affected by the type of sport played by the athletes.^6,7^ Specifically, contact sports have been shown to have worse sports outcomes than non-contact sports.^6,7^ Furthermore, most of these studies use the term “contact sports” to include different types of sports and evaluate return to sports in a global way without considering the return to each specific sport separately.^8^ In addition, no study has been found in which return to sports after an ABR was evaluated according to the playing position in rugby.

Regarding recurrences after an ABR, studies showed a recurrence rate between 4% and 51%.^9^ Furthermore, several risk factors, such as playing a contact sport have also been documented.^9,10^ Specifically in rugby union, several observational studies have identified certain positions at risk for traumatic anterior shoulder dislocation.^11^ However, apart from some contradictory findings in those studies, no study could be found evaluating the playing position as a risk factor for recurrences after an ABR in rugby players.

As each rugby player is subject to different and unique demands imposed on the shoulder, the likelihood to have different sports and surgical outcomes after ABR may be affected by their position on the field. Hence, a thorough understanding of the influence of a player’s position on sports and surgical outcomes after an ABR can facilitate coaches and medical staff to establish much more precise prevention and rehabilitation strategies.^2,3^

Therefore, the purpose of our study was to investigate the influence of the different rugby playing positions on return to sports, functional outcomes, and recurrences after an ABR.

The hypothesis of our study was that return to sports, functional outcomes, and recurrences after ABR would vary significantly among the different rugby playing positions.

## Methods

This was a retrospective cohort study. Between January 2010 and December 2018, 120 athletes who were competitive rugby players underwent ABR at our institution.

The inclusion criteria for this study were: competitive rugby players (regular sports with competitions and practices at least 2 times/wk),^12^ a minimum follow-up period of 24 months, at least one instability episode (defined as a dislocation with spontaneous reduction or complete dislocation requiring a reduction).

Exclusion criteria were: large bony Bankart lesions (bony defects of >20% on the anteroinferior portion of the glenoid), engaging Hill-Sachs lesions, humeral avulsion of the glenohumeral ligament lesions, associated superior labral from anterior to posterior (SLAP) lesions, posterior labral tears, rotator cuff injuries, or previous surgery on the same shoulder.

**Figure 1.**
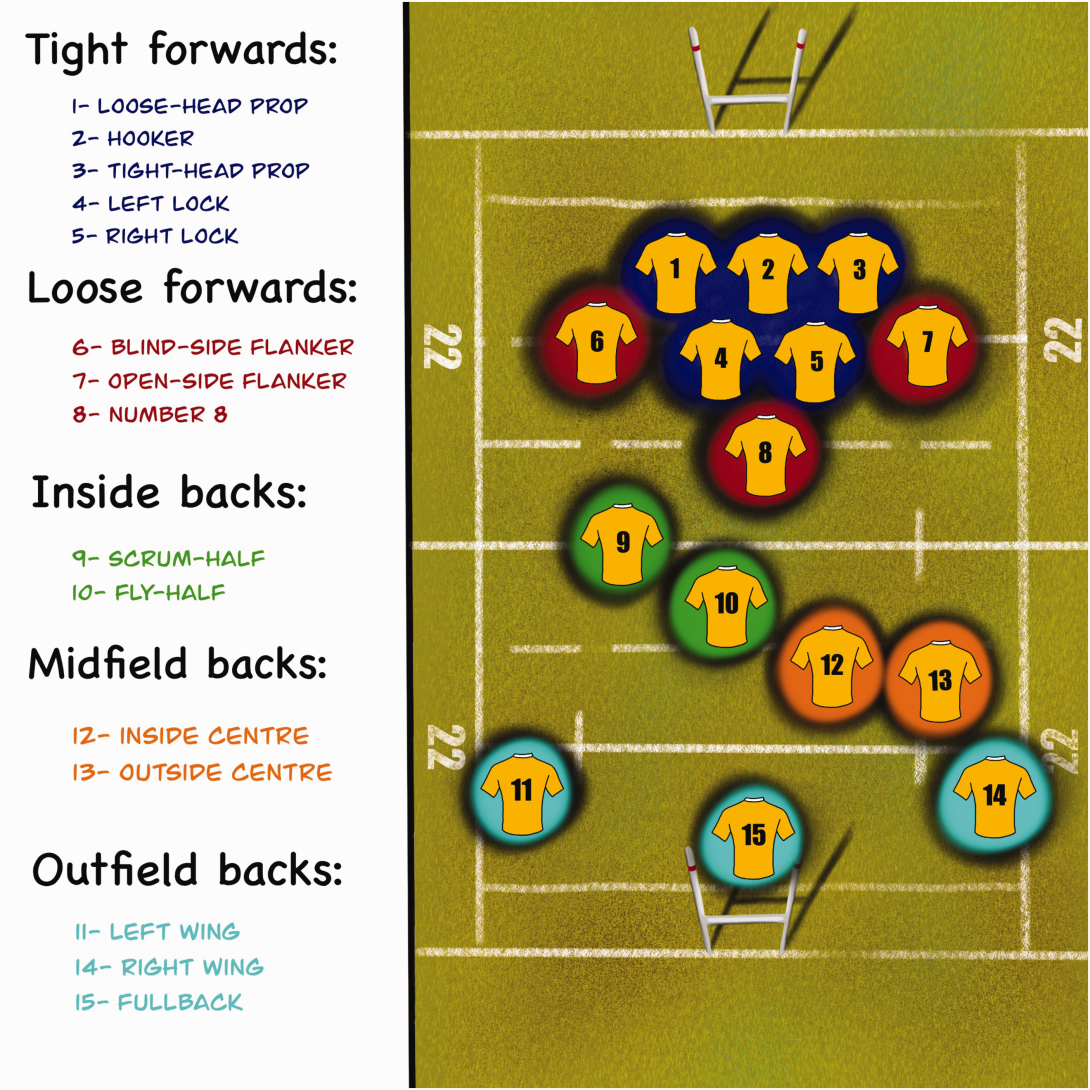
Rugby player’s on-field positions.

Rugby player’s positions were classified into five groups according to their on-field position.^13^ (Figure 1). The mechanisms of injury were classified as “try scorer”, “tackler”, “direct impact”, and “poach position”.^14,15^

The study protocol was approved by the local ethics committee of our institution (No. IRB 00010193), and all patients provided written informed consent to participate in this investigation.

### Evaluation

Preoperative and postoperative evaluations consisted of a patient-based questionnaire and a physical examination performed by a shoulder fellow who did not participate in the surgical procedure. Instability was evaluated with apprehension and relocation tests. Radiography and magnetic resonance imaging were performed in all cases. If, during these studies, bony defects were suspected, computed tomography was ordered to evaluate the magnitude.

The Rowe score was used as a global outcome measure.^16^ Shoulder-dependent sports ability was measured with the Athletic Shoulder Outcome Scoring System (ASOSS).^17^ Patients were contacted by telephone and then examined at a minimum follow-up of 24 months. Patients were also asked if they had been able to practice their previous sports and if they had been able to perform them at the same level as before the dislocation. All surgery-related recurrences, complications, and reoperations were documented. We defined recurrence as the presence of a dislocation, subluxation or apprehension during the follow-up physical examination that limited daily activities or sports.

### Surgical technique

The surgical technique for all of the cases in this series was an anterior arthroscopic stabilization performed in the lateral decubitus position with combined general endotracheal and regional anesthesia. All athletes underwent primary arthroscopic anterior glenohumeral stabilization surgery for anterior shoulder instability using a knotted anchor technique with simple sliding knots. In all cases we used biodegradable anchors with double suture. After complete liberation and release of the capsulolabral ligament beyond the 6-o’clock position, the labral edge was debrided. Then, the anterior and inferior glenoid rim and neck were abraded with a shaver. Typically, three anchors with No. 2 nonabsorbable sutures (CrossFT™ ConMed) were placed on the cartilage edge of the glenoid surface, mean 3.2 (range 2–4). The first one was placed in the inferior area of the anterior glenoid rim below the 5-o’clock position. Additional anchors were placed in a similar manner at both the 3- and 4-o’clock positions. No patients in this series were treated with a posterior-inferior capsulolabral repair, rotator interval closure, SLAP repair, or remplissage.

### Rehabilitation

A standardized postoperative physical therapy and rehabilitation program was used. The arm was supported in a sling for 4 weeks. After 1 week, supervised gentle physical therapy consisting of gradual passive range of motion (ROM) was begun. Active-assisted ROM exercises were started 2 weeks after surgery. When the patient could perform active forward elevation above the shoulder level, strengthening exercises were started. Running was authorized at 8 weeks. Return to sports was allowed when the patient was pain free without apprehension, full shoulder ROM had been achieved, and shoulder strength was near the same as before the injury.

### Statistical analysis

Continuous variables are presented as means ± SDs, and categorical variables are presented as absolute and relative frequencies. To compare the proportions of the categorical variables between the groups of rugby on-field positions, the chi-square test or the Fisher exact test was used according to their assumptions. One-way ANOVA or Kruskal–Wallis test was used to compare the differences between the medians between the groups according to their assumptions. A logistic regression model was used to evaluate the association between the on-field position and recurrences at follow-up. The crude and adjusted odds ratios (OR) are presented with their 95% confidence intervals (95% CI) and their *p* values. The statistical analysis was performed using STATA version 13 (Stata Corp). *p* values under 0.05 were considered statistically significant.

## Results

**Figure 2.**
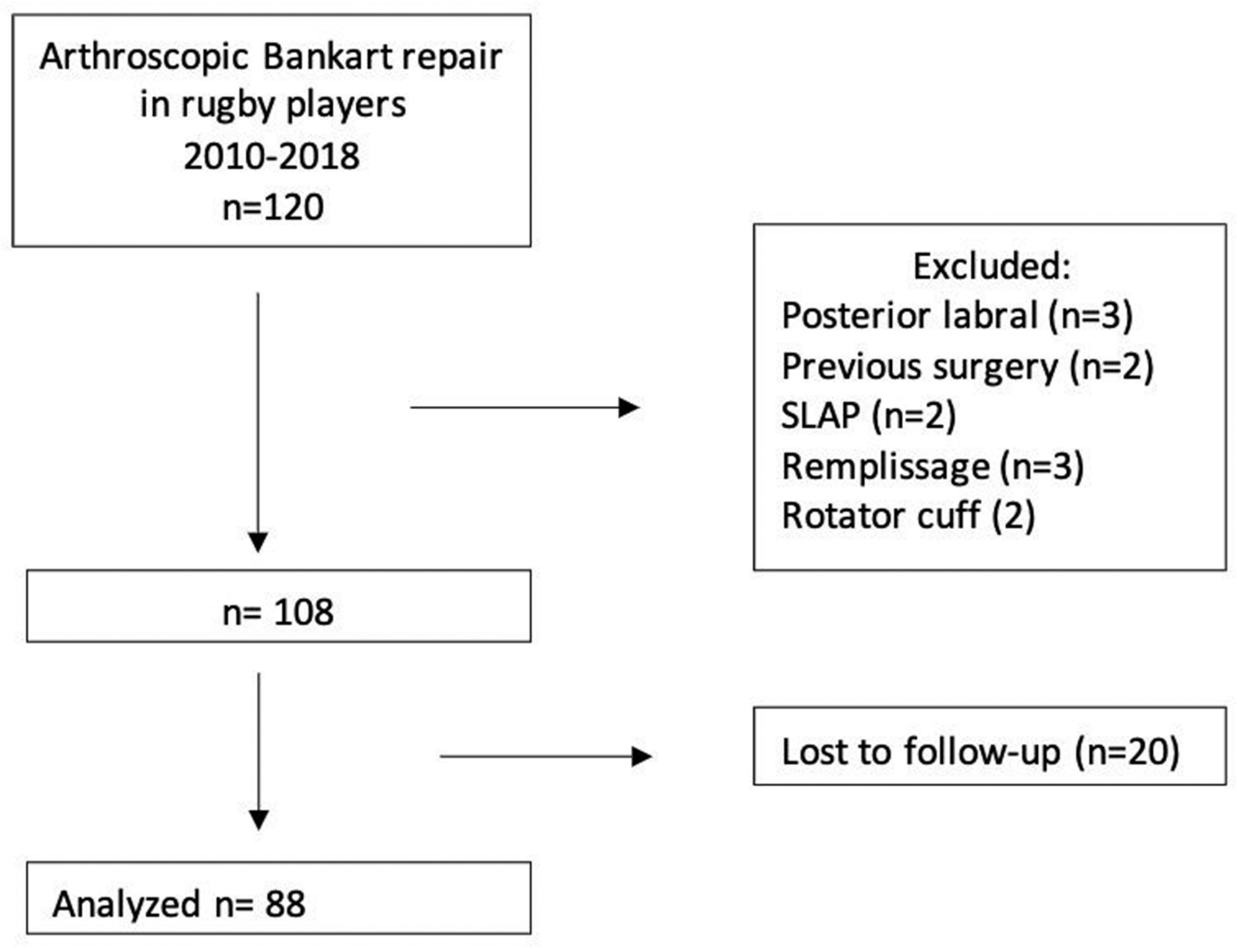
Flow chart demonstrating the patients selection process.

There were 120 consecutive patients who required arthroscopic stabilization following an on-field Rugby injury between January 2010 and December 2018. Of these, we excluded 12 patients because they did not meet the inclusion criteria and 20 because of an incomplete minimum follow-up. Thus, the final analysis entailed 88 patients (Figure 2).

All the included patients were males. The mean age at the time of surgery was 21.3 years (SD 4.8), and the mean follow-up period was 59.5 months (SD 30.5). The dominant arm was involved in 41 cases (46.5%). The baseline characteristics of the included patients are shown in Table 1. The distribution of the players according to their playing positions and the mechanisms of injury are described in Table 2.

**Table 1.** Comparison of baseline characteristics between groups.

Variable	Tight forwards (*n* = 19)	Loose forwards (*n* = 24)	Inside backs (*n* = 18)	Midfield backs (*n* = 12)	Outfield backs (*n* = 15)	*p* value
Age at surgery, *y* mean (SD)	21.8 (6.6)	21.2 (4.2)	20.7 (4.1)	21.9 (5)	21.2 (4.1)	0.9833
Follow-up, mean (SD)	60 (30.9)	60 (31.4)	60 (22.9)	64 (33.6)	54.1 (36.8)	0.9492
Dominant involved, % (*n*)	52.6% (10)	54.1% (13)	44.4% (8)	33.3% (4)	40% (6)	0.752
*n* anchors, mean (SD)	2.8 (.3)	2.9 (.2)	2.8 (.3)	2.9 (.2)	2.9 (.2)	0.7509
BMI, mean (SD)	28.5 (4.3)	26.4 (2.7)	25.1 (1.8)	25.6 (2.6)	24.2 (1.3)	0.0078
Rowe Pre, mean (SD)	41 (14)	41.6 (12.5)	42.5 (10.7)	43.3 (15.5)	39.6 (17.1)	0.9656
ASOSS Pre, mean (SD)	53.5 (4)	52.9 (2.6)	53 (2.3)	53.1 (1.9)	53.8 (2.3)	0.4914

SD: standard deviation; ASOSS: Athletic Shoulder Outcome Scoring System.

**Table 2.** Distribution of on-field positions and mechanisms of injury between groups.

Variable	Tight forwards	Loose forwards	Inside backs	Midfield backs	Outfield backs	*p* value
On-field position % (*n*)	21.6% (19)	27.3% (24)	20.4% (18)	13.7% (12)	17% (15)	
Mechanism of injury % (*n*)						
Tackler	47.4% (9)	50% (12)	88.9% (16)	58.3% (7)	60% (9)	0.064
Direct impact	52.6% (10)	37.5% (9)	11.1% (2)	41.7% (5)	20% (3)	
Poach position		8.3% (2)			13.3% (2)	
Try scorer		4.2% (1)			6.7% (1)	
Moment of injury:						
Match	84.2% (16)	87.5 % (21)	72.2% (13)	83.3% (10)	93.3% (14)	0.593
Training	15.8% (3)	12.5 % (3)	27.8% (5)	16.7% (2)	6.7% (1)	

Overall, 73.8% (65) returned to rugby and 23% (21) returned to play other sports (8 gym, 7 soccer, 2 tennis, 2 CrossFit, 1 triathlon, 1 lacrosse). Moreover, the reasons for sport cessation in those who did not return to rugby were all independent from their shoulder function (fear of reinjury, lack of confidence, age, lack of time). From those who returned to rugby, 60% (53) returned at the same level as before the injury. The mean time to return to rugby was 6.8 (SD 1.6) months, respectively. The comparison of sports outcomes is shown in Table 3.

**Table 3.** Comparison of sports outcomes between groups.

Variable	Tight forwards	Loose forwards	Inside backs	Midfield backs	Outside backs	*p* value
Return to rugby, % (*n*)	68.4% (13)	75% (18)	77.8% (14)	75% (9)	73.3% (11)	0.983
Return same level, % (*n*)	58.3% (7)	94.4% (17)	100% (14)	88.9% (8)	63.6% (7)	0.007
Time to return to rugby, months	7.2	6.4	7.6	6.4	6.2	0.3373

The Rowe score and ASSOS score showed statistical improvement after operation (*p* < 0.001). Specifically, the Rowe score increased from a preoperative mean of 41.5 (13.7) to a postoperative mean of 92.2 (SD 13.5; *p* < 0.001). The ASOSS score improved significantly from a preoperative mean of 53.2 (SD 2.7) to a postoperative mean of 92.1 (SD 13.4; *p* < 0.001). Comparative postoperative functional outcomes between groups are shown in Table 4.

**Table 4.** Comparison of functional outcomes between groups.

Variable	Tight forwards	Loose forwards	Inside backs	Midfield backs	Outfield backs	*p* value
Final Rowe, mean (SD)	86.8 (16.5)	95.6 (9.8)	95.8 (8.7)	94.1 (11.8)	87.6 (18.1)	0.1583
Final ASOSS mean (SD)	87.1 (15.9)	95.4 (11.5)	95.7 (8.2)	93 (10.4)	88.3 (17.9)	0.3483

The recurrence rate was 32.3% and the complication rate was 4.6%. Sixteen percent of the patients underwent revision surgery, all due to traumatic episodes during competition or training. Comparative recurrence rate, complication rate, and revision rate between groups are shown in Table 5. Regarding the recurrence rate in tight forwards 80% were hookers and 20% were locks. Regarding the recurrence rate in outside backs, 100% were fullbacks. The crude and adjusted ORs with 95% CI of the on-field positions for recurrent instability are reported in Table 6.

**Table 5.** Comparison of recurrences, complications, and revision rates between groups.

Variable	Tight forwards	Loose forwards	Inside backs	Midfield backs	Outfield backs	*p* value
Recurrence in rugby, % (*n*)	61.5% (8)	16.7% (3)	21.4% (3)	11.1% (1)	54.5% (6)	0.019
Complications, % (*n*)	5.2% (1)	4.1% (1)	0% (0)	16.6% (2)	0% (0)	0.244
Revisions, % (*n*)	26.3% (5)	4.1% (1)	5.5% (1)	8.3% (1)	40% (6)	0.015

**Table 6.** Univariate and multivariate binary logistic analysis of on-field positions for recurrent instability adjusted by age, BMI, and return same level.

	Crude OR	95% CI	*p* value	Adjusted OR	95% CI	*p* value
Midfield backs	Ref					
Inside backs	2.1	0.19–25	0.531	3.7	0.25–56.8	0.337
Outside backs	9.6	0.87–105.1	0.064	8.1	0.57–114.7	0.121
Tight forwards	12.8	1.2–135.5	0.034	30.1	1.6–548.3	0.021
Loose forwards	1.6	0.14–18	0.704	2.6	0.19–37.7	0.470

Ref: reference category.

## Discussion

This study has four main findings. First, significant differences were found regarding the level achieved after surgery between groups. Indeed, it significantly varied ranging from 58.3% to 100%. Specifically, 58.3% of the tight forwards and 63.6% of the outside backs returned to the same level. Second, even though we did not find a significant statistical difference between the groups, the tight forwards and outside backs showed the lowest functional outcome scores of all groups. Third, we found a significant difference in the recurrence and revision rates between groups. Notably, the group from the tight forwards and outside backs showed higher recurrence and revision rates. Fourth, we found that the tight forwards and outside backs were 12.8 and 9.6 times more likely to have a recurrence than the midfield backs, which was the group with the least recurrence rate.

Generally, ABRs have demonstrated a return to sports rate ranging from 56% to 98%.^18^ A recent meta-analysis by Memon et al.^18^ reported that 82% of competitive athletes returned to sports with 88% returning at preinjury level. Similarly, another metaanalysis by Ialenti et al.^19^ reported that 71% of patients returned to sports at the same level of play. However, several authors have reported that contact and overhead athletes can yield less favorable results.^17,20^ Stein et al.^17^ have found that G3 and G4 athletes returned to inferior levels compared to G1 and G2 athletes. Cho et al.^6^ reported only 65% rate of complete return to preinjury levels in collision athletes. Similarly, Ranalletta et al.^21^ showed that only 60% of martial arts athletes could achieve the same level before surgery. Furthermore, all of these results were reported in a general way, not considering the specific return to each specific sport separately. Therefore, by knowing the exact return to sports rate of each sport, physicians could accurately inform the athletes about what results they should expect after surgery. Moreover, no study could be found reporting return to sports rates after an ABR according to the playing position of the athletes. Notably, in our study, we found that 73.8% returned to rugby and 23% returned to other sports. Moreover, from those who returned to rugby, 60% achieved the level they had before surgery. However, the level achieved after surgery by our rugby players varied significantly according to their playing position. Specifically, we found that the tight forwards and outside backs returned to an inferior level compared to the other groups. This finding could be explained in several ways. Regarding the tight forwards, they are constantly involved in high intensity shoulder demanding activities, such as tackles, scrums, mauls, rucks, and line outs. Even though these activities are common for all of the forwards, in the scrum, Martin and Beckham^22^ described that the front rows produce 40–51% of the average or maximum sustained pack force, locks produced 31–33% of these forces, and just 18–27% were produced by the loose forwards. Furthermore, the tight forwards are usually in charge of the line out, which is an overhead throwing activity where the shoulder is constantly demanded.^23^ Regarding the outside backs, first of all, they have a very physically demanding position, in which they are expected to use great velocities to gain territory while attacking.^24^ Similarly, Lindsay et al.^25^ showed that the outside backs covered more distance at >20 km/h than the other positions. Second, the outside backs have a different type of tackle compared to the other positions, they usually cover greater spaces at high velocities before getting involved in a tackle. Therefore, fatigue accumulates and consequently their tackle technique is compromised.^24,26^ This could be explained by the fact that fatigue has been demonstrated to have an influence on shoulder position sense and tackle technique.^26^ Finally, this finding is important because it could help medical staff to develop a position-specific training when rehabilitating an injured player to maximize the level achieved after surgery.

In the literature, there are excellent functional outcome scores reported after an ABR.^4,27^ Larrain et al.^28^ found 94.9% of good or excellent results in their 121 rugby players who underwent an ABR. Similarly, we found a final Rowe and ASOSS score of 92.2 (SD 13.5) and 92.1 (SD 13.4), respectively. Finally, even though we did not find a significant statistical difference between the groups, the tight forwards and the outside backs showed the lowest shoulder functional outcome scores.

In the current literature, recurrence rates after an ABR ranges from 4% to 51%.^9^ Contact and collision sports have been described as contributing factors to the risk of recurrence. A recent systematic review by Alkaduhimi et al.^29^ reported that the recurrence rates for collision athletes vary from 5.9% to 38.5% compared to 0% to 18.5% for non-collision athletes. Moreover, they found that collision athletes have an increased risk of 8.09 for sustaining a recurrence episode than non-collision athletes. Furthermore, several studies have reported recurrence rates after an ABR in rugby players. Castagna et al.^30^ reported a recurrence rate of 33.3% in their rugby players. In line with this, a study by Nakagawa et al.,^31^ in which risk factors for postoperative recurrence of instability after an ABR were investigated, found a recurrence rate of 33.3% in their rugby athletes. Finally, Torrance et al.^9^ studied the recurrence rate after an ABR in adolescent rugby and contact athletes finding a recurrence rate of 51%. Similarly, in our series, we found a recurrence rate of 32.3%. Although several observational studies have reported some positions at risk of injury, these results remain controversial.^2,11^ Moreover, no study could be found with a focus on recurrence after an ABR in rugby athletes according to their playing position on the field. In our study, we found significant different recurrence rates between the groups. Specifically, the tight forwards (80% hookers) and outside backs (100% fullbacks) reported higher recurrence rates than the other groups. Moreover, we found that the tight forwards significantly increased the odds of recurrence after an ABR by 12.8 compared to the midfield backs. Although we did not find a statistically significant increase of the odds in the outside backs, they show a considerable increase of 9.6. Another interesting finding was that the significantly increased odds of the tight forwards were maintained after adjusting for age, BMI, and level achieved after surgery. Similarly, a recent study by Montgomery et al.^14^ showed that the hookers and fullbacks had the highest number of shoulder dislocations. This finding could be explained in several ways. Regarding the tight forwards, they are more likely to be involved in contact events than the backs.^13^ Indeed, they are generally involved in highly physically demanding activities such as tackles, scrums, rucks, and mauls. Although all forwards are typically involved in these events during the match, Quarrie et al.^13^ reported that the physical demands on players by scrums vary with position. Specifically, the front row undergoes heavy loads in each scrum, producing 3290 N, while the full scum produced 3370 N, indicating that the front row alone could produce 98% of the scrum force.^22^ Moreover, the vulnerability of the hooker in the scrum has been attributed to a number of factors: first, the wrapping of their arms around the props in the scrum with the effect that he or she cannot control or dissipate forces of engagement; second, the reliance on the props for support during engagement and formation; and third, the inability to adjust upper body position to react to improper engagement.^32^ Finally, Kawasaki et al.^2^ also demonstrated that the front row players are more likely to experience a shoulder injury. On the other hand, although the outside backs are less likely to make tackles than the forwards, they generally travelled greater distances before being involved in a tackle, and therefore their tackling technique is implicated in their injuries.^24^ Player fatigue was implicated in tackle-related injuries and was associated with deterioration in the tackle technique. In line with our findings, Sundaram et al.^11^ showed that full backs are more likely to get injured than the wings. The higher risk of anterior instability recurrences in the fullbacks than wings can be attributed to the anthropometric and physiological variations between positions, the physical workload, and the use of incorrect or inefficient techniques.^24^ The wing is the position that makes the lowest number of tackles, spends the highest proportion of match time completing low intensity activities, has the longest recovery times and has the fastest 40 m sprint time, which was considered a protective factor against injury in Rugby league.^24^ Finally, Gabbet et al.^33^ showed that the outside backs were the only positions that were more likely to get injured while attacking, especially because they are often tackled by three and sometimes four defenders. Therefore, by knowing the positions that are at more risk of sustaining a recurrence after an ABR, physicians could generate prevention strategies for those positions with the aim to reduce the redislocation rate in rugby players after an ABR.^34^

The ABR is considered a safe and effective procedure with low complication rates.^35^ Indeed, we found a total complication rate of 4.6%; and we did not find significant differences between the groups. Concerning revision rates, a recent systematic review by Murphy et al.^27^ found an overall revision rate of 17% after an ABR. Similarly, we found a total revision rate of 15.9%. However, we did find significant differences between the groups. This finding was suspected to be found as all the revision surgeries were due to recurrent instability episodes.

This work has some limitations which should be mentioned. First, as a retrospective study, it has all the limitations inherent to this kind of study. Second, we did not have a control group to compare our results. It would have been useful to have a control group operated with another surgical technique, such as the Latarjet procedure, since in this way it could have been evaluated whether the results of this study, regarding the influence of the playing position on the sports and surgical outcomes, were due to the surgical technique elected or whether the results are maintained despite changing the surgical technique. Finally, it would have been interesting to evaluate the risk of each position (15 rugby players) on sports and surgical outcomes after an ABR. However, we did not have the sufficient athletes to do it. Moreover, given the low number of cases per group, there may have been a beta error in data analysis, which may have led to not seeing a statistical difference. Nevertheless, we believe that this study has a sufficient number of patients in each of the five groups included to demonstrate our hypothesis.

## Conclusion

The playing position significantly influenced return to sports and recurrences after an ABR in competitive rugby athletes. Specifically, the tight forwards and outfield backs have returned to a lower level than they had before surgery and showed the lowest functional outcomes. Moreover, these same groups showed a significant increase in recurrences and revisions rates than the other groups.
